# Supplementary material for: Neurophysiological Correlates of Top-Down Phonological and Semantic Influence during the Orthographic Processing of Novel Visual Word-Forms
Source: Brain Sci. 2020 Oct 9;10(10):717. doi: 10.3390/brainsci10100717 (PMC7601445; doi:10.3390/brainsci10100717)
Supplement: Supplementary file 1 [file brainsci-10-00717-s001.pdf]

Supplementary material

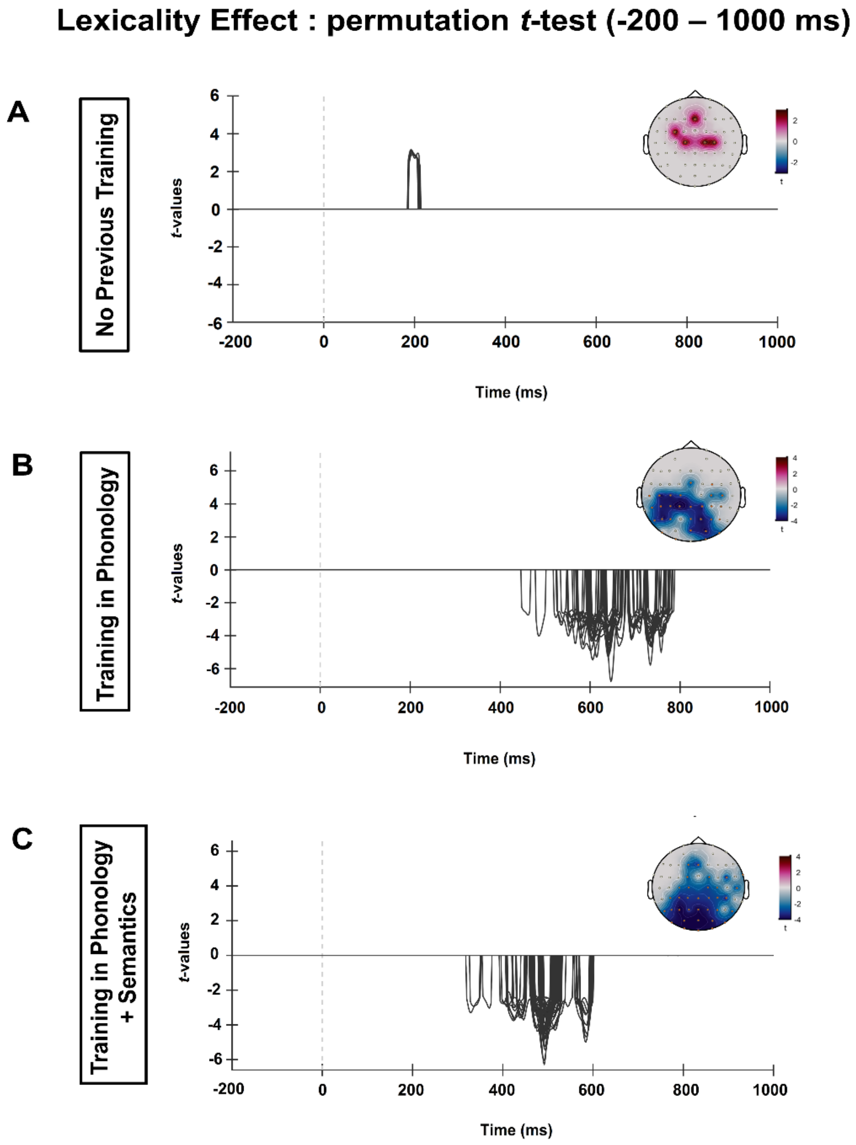

Supp. Figure 1. Results from permutation analyses. A *t*-test contrasting known and trained words was carried out across Experiments 1, 2 and 3 (panels A to C, respectively). Known and trained words were contrasted by means of paired *t* tests, including a total of 1,000 permutations computed for each sample point across the whole ERP segment, ranging from -200 to 1000 ms. For each comparison at each sample point, *t*-values reaching statistical significance are displayed, together with the topographical map showing the scalp distribution and electrodes in which comparisons reached significance (only those differences maintained for a minimum of 20 ms, involving at least 3 sensors and below alpha level = 0.025 were considered significant).
